# Utility and greenness appraisal of nuclear magnetic resonance for sustainable simultaneous determination of three 1,4-benzodiazepines and their main impurity 2-amino-5-chlorobenzophenone

**DOI:** 10.1038/s41598-023-48416-7

**Published:** 2023-11-30

**Authors:** Nermeen A. Qandeel, Amal A. El-Masry, Rania El-Shaheny, Manal Eid, Mohamed A. Moustafa

**Affiliations:** 1 Department of Medicinal Chemistry, Faculty of Pharmacy, Mansoura University, Mansoura, 35516 Egypt; 2 Department of Pharmaceutical Analytical Chemistry, Faculty of Pharmacy, Mansoura University, Mansoura, 35516 Egypt

**Keywords:** Chemical safety, Green chemistry, Medicinal chemistry

## Abstract

A robust, stability-indicating, and eco-friendly proton nuclear magnetic resonance (^1^H-qNMR) method was developed for the concurrent determination of three 1,4-benzodiazepines (BDZs), namely diazepam (DZP), alprazolam (ALP), and chlordiazepoxide (CDP) and their common impurity, synthesis precursor, and degradation product; 2-amino-5-chlorobenzophenone (ACB). In the present method, a novel approach was developed for composing a green and cost-efficient solvent system as an alternative to the common NMR organic solvents utilizing 0.3 M sodium dodecyl sulfate prepared in deuterated water. The conducted method is characterized by simplicity with no need for sample pretreatment or labeling. Phloroglucinol was used as an internal standard. The chosen signals for the determinations of ALP, CDP, DZP and ACB were at 2.35 ppm (singlet), 2.84 ppm (singlet), 3.11 ppm (singlet), and 6.90 ppm (doublet of doublet), respectively. The proposed method possessed linearity over the concentration range of 0.25–15.0 mg ml^−1^ for DZP, ALP, CDP and of 0.5–25.0 mg ml^−1^ for ACB with LOD values of 0.06, 0.03, 0.07 and 0.16 mg ml^−1^ respectively, and LOQ values of 0.18, 0.09, 0.21 and 0.49 mg ml^−1^, respectively. Accuracy of the method was evidenced by excellent recovery% (99.57–99.90%) and small standard deviation (≥ 1.10) for the three analyzed drugs. Intra- and inter-day precision were determined with coefficient of variation ranging from 0.12 to 1.14 and from 0.72 to 1.67, respectively. For the studied compounds, appraisal of the method greenness was achieved via four approaches: Analytical Eco-Scale, Green Analytical Procedure Index (GAPI), Analytical greenness metric (AGREE), and RGB Additive Color Model. The results proved that the proposed method has the privilege of being a green analytical method.

## Introduction

1,4-Benzodiazepines (BDZs) are a major class of agents used for short- and long-term treatment of anxiety and related disorders. These drugs have been used also as anti-epileptics, anxiolytics and for treatment of sleeping disorders due to their sedative effect on the central nervous system. They bind to an allosteric site on gamma-aminobutyric acid (GABA) receptor leading to potentiation of the inhibitory effect of GABA neurotransmitter through increasing the chloride conductance^1–3^.

The continuing popularity of BDZs is due to their constant and reliable efficacy for most symptoms of anxiety, comparatively good tolerability, rapid onset of action, and the recognition that newer antidepressants have not been as useful for these conditions as they had initially appeared to be. BDZs addiction risk has been exaggerated and they may be used as first-line pharmacotherapy as a safe option for the long-term treatment of many patients with anxiety and related disorders. Unfortunately, benzodiazepines are present in the illicit market and used by addicted individuals and leading to increased suicide rates and road insecurity^1–3^.

**Figure 1:**
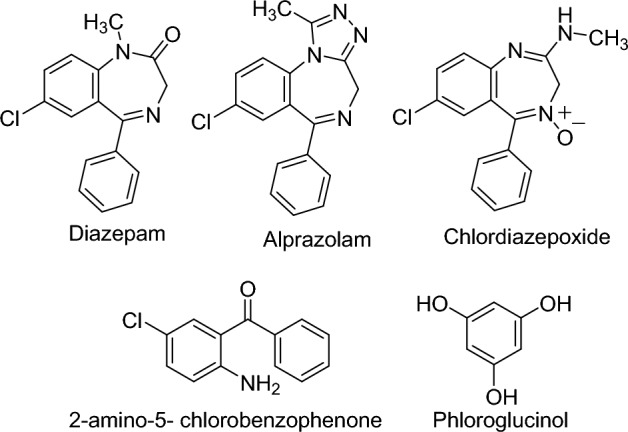
Chemical structure of analyzed compounds and the internal standard.

As completely synthetic drugs, the presence of synthesis precursors as impurities in BZDs is a dilemma and must be detected to verify the products that fulfill the criteria of quality control^4^. 2-Amino-5-chlorobenzophenone (ACB) is categorized as a precursor in the synthesis of BZDs, thus it is a common impurity in many BDZs^5^. Consequently, it is an important issue to develop a reliable method for detection of ACB in BDZs and simultaneous determination of both BZDs and ACB. Diazepam (DZP), chemically known as 7-chloro-1,3-dihydro-1-methyl-5-phenyl-2H-1,4-benzodiazepin-2-one^3^ (Fig. 1) is a long-acting BDZ that is used for the treatment of epilepsy, anxiety, muscle spasms and for alcohol withdrawal^6^. On the other hand, alprazolam (ALP), (8-chloro-1-methyl-6-phenyl-4*H*-*s*-triazolo[4,3-a][1,4]benzodiazepine^3^, Fig. 1), is a moderately-acting BDZ used for treatment of anxiety disorders and panic attacks^7^. Chlordiazepoxide (CDP), chemically named as 7-chloro-2-(methylamino)-5-phenyl-3*H*-1,4-benzodiazepine-4-oxide^3^ (Fig. 1) is another long-acting BDZ due to its active metabolite *N*-desmethylchlordiazepoxide and also used in cases of moderate to severe anxiety and alcohol withdrawal^8, 9^. The three BDZs are ACB derivatives. Yet, reviewing the analytical literature revealed the lack of a reliable method to analyze the three BDZs with their main impurity and synthesis precursor ACB.

Several analytical methods were implemented for the determination of these drugs either alone or in combination. For example, liquid chromatography-tandem mass spectrometry (LC–MS/MS) methods^10, 11^ have been applied for the determination of some combinations of these BDZs. DZP and ALP were determined in urine by LC–MS/MS^10^ with a detection limit (LOD) of 10 ng ml^−1^, and DZP, ALP and CDP have been determined in human serum by LC–MS/MS with LOD of 0.6–2.0 ng ml^−1^. In addition, ultra-high performance liquid chromatography-mass spectrometry (UHPLC-MS) has been applied for the determination of group of BDZs including DZP and ALP in human hair samples within the linearity range of 0.01–6.9 ng mg^−1^ and LOD of 0.003–0.025 ng mg^−1^^12, 13^. Another UHPLC-MS/MS^13^ was devoted to the determination of DZP and ALP in antemortem and postmortem whole blood samples with LOD of 8.5 and 0.46 ng ml^−1^, respectively. Furthermore, a gas chromatography-mass spectrometry (GC–MS) method^14^ has been applied for the determination of DZP, ALP, and CDP in human plasma with LOD within the range of 0.52–58.47 ng ml^−1^ and linear range from 5.0 to 2000 ng ml^−1^. Furthermore, some electrochemical methods have been applied for the determination of DZP in tablets^15^, human urine^16^, and human hair^17^. In addition, DZP has been determined also in tablets and human urine by spectrophotometric methods^15, 16^. Furthermore, some high performance liquid chromatography (HPLC)^17–20^ have been applied for the determination of these BDZs in different matrices. For example, HPLC method was applied for the determination of DZP in rat plasma and brain tissue within the concentration range of 5.0–250.0 ng ml^−1^ with LOD of 1.0–1.7 ng ml^−1^. Furthermore, HPLC methods have been applied for the determination of ALP^20^, DZP and ALP^22^ in human serum with LOD of 0.01 ng ml^−1^, and DZP and CDP^23^ in human urine with LOD of 200–400 ng ml^−1^. Besides, HPLC method^5^ determined CDP and ACB in tablet formulation over the concentration ranges of 1.0–100.0 and 2.0–40.0 µg ml^−1^, respectively.

However, these methods require hazardous chemicals such as formic acid and nitric acid, plenty of organic solvents such as methanol, 2-propanol, heptane, and acetonitrile, isotope labeled standards, and chemical derivatization. Moreover, some of these methods require several steps such as hydrolysis and extraction of the active drug to be analyzed. Meanwhile, these methods were not validated as stability-indicating assays. This motivated us to develop the first stability-indicating quantitative/qualitative green ^1^H-qNMR method for simultaneous determination of three BDZs including: DZP, ALP and CDP and their impurity/degradation product ACB while conserving the main principles of green analytical chemistry.

Green chemistry is a concept that is widely applied in many fields even at the molecular level. Applying the green concept in chemical analysis improves the analytical method’s environmental and economic aspects. This can be achieved by using eco-friendly solvents and avoid using hazardous substances or procedures^21–23^. The utility of ^1^H-qNMR in the pharmaceutical analysis field has many benefits owing to the advantages of being highly precise and accurate in determining the purity of pharmaceutical compounds. ^1^H-qNMR is a modern concept that involves the quantitation of certain substance by detecting the area under the peak of the specific signal, which is directly proportionally to its concentration^24^. Though, most of the reports on ^1^H-qNMR applied CDCl_3_ or DMSO-d_6_. Taking in consideration that these solvents entail harmful adverse effects on human health, they occupy inferior order in the solvent selection guide, and they are expensive, it is advisable to replace them with greener and safer solvents^25^. In this vein, sodium dodecyl sulfate (SDS) is a widely used surfactant due to its low price and superior dissolution capacity. Therefore, it has been employed in various green analytical methods due to its green properties^26–29^. Therefore, in this work, SDS has been used to enhance the solubility of the practically water-insoluble analyzed compounds^30^ in deuterated water by its micellar effect while maintaining the green aspects of the proposed analysis method, omitting the organic solvents, and enhancing the cost-efficiency of the developed method.

In the present study, a new quantitative and qualitative green ^1^H-qNMR method is used for the first time for quantification of three BDZs including: DZP, ALP and CDP in different dosage forms utilizing eco-friendly solvent as deuterated water in the presence of SDS. Furthermore, it is employed for stability-indicating assay for detection, identification, and quantification of ACB impurity in these BDZs to ensure the quality of the tested dosage forms.

## Experimental

### Apparatus and condition

The concurrent assays of quadrilateral mixture of DZP, ALP and CDP in presence of ACB impurity were carried out by ^1^H-qNMR. Spectra were recorded at 400 MHz using a Bruker advance III spectrometer through the following acquisition and elaboration parameters: time of acquisition (4.08 s), spectral width (20.02 ppm), pulse angle (30°), frequency offset (6.175 ppm), pulse width (4.5 μs), sample temperature (20 °C), number of scans (256), dummy scans (2), relaxation delay time (20 s), sample spin (on) and data points (65,536). The NMR spectra revealed singlet at 2.35 ppm referring to ALP, singlet at 2.84 ppm referring to CDP and a singlet at 3.11 ppm referring to DZP, while one doublet of doublet at 6.91, 6.90, 6.89, 6.88 (6.90) ppm referring to ACB impurity. A final detectable signal is a singlet one at 5.65 ppm which refers to phloroglucinol (PGC), the used internal standard in this study. The spectra were analyzed using auto phase correction, 0.3 Hz exponential line-broadening function, integration, and baseline correction.

The comparison spectrophotometric method was performed with a Shimadzu UV-1601PC UV-1601PC Spectrophotometer (Kyoto, Japan) with a one cm matched quartz cuvettes. Fast scan speed was adjusted in all recorded absorption spectra. Eumax ultrasonic bath (model SS 101 H 230, USA) was used for sonication. A 0.2 µm cellulose acetate-driven syringe filter (LMS Co., Tokyo, Japan) was used for filtration of the prepared solutions.

### Materials and reagents

Pure forms of DZP (99.87% ± 1.10, Spectrophotometric method^31^), ALP (99.85% ± 2.05, spectrophotometric method^32^), and CDP (99.93% ± 0.65, spectrophotometric method^33^) were kindly donated by (Nile Co. for pharmaceuticals and chemical industries, Cairo, Egypt), (Global Napi Pharmaceuticals, Egypt) and (Eva Pharma Co., Cairo, Egypt), respectively. Additionally, ACB, n-propanol, SDS and the internal standard PGC were purchased from Sigma Aldrich Co., U.S.A. Lactose, talc, and starch were purchased from Chemi-Pharm for Pharmaceutical Industries, Giza, Egypt. The deuterated solvents used in this study (deuterium oxide (deuterated water, D_2_O)) and (DMSO-*d*_*6*_) were purchased from Cambridge Isotope Laboratories. Valinil tablets (The Nile Co. for pharmaceuticals and chemical industries, Cairo, Egypt) containing 5.0 mg DZP per tablet and Restolam tablets (Global Napi Pharmaceuticals, Egypt) containing 1.0 mg ALP per tablet were purchased from a local pharmacy.

### Preparation and analysis of standard solutions

Standard stock solutions of DZP, ALP and CDP (25.0 mg ml^−1^ for each) were prepared by transferring 0.125 g of each of them separately into three 5-ml volumetric flasks. The drugs were dissolved in 0.3 M SDS/D_2_O solution, and the volumes were completed to the mark using the same solvent. Moreover, standard stock solutions of ACB and PGC (50.0 mg ml^−1^, 100.0 mg ml^−1^, respectively) were prepared by dissolving 0.25 g and 0.50 g of ACB and PGC, respectively in two different 5-ml volumetric flasks. The volumes were completed to the mark using 0.3 M SDS/D_2_O solution. All volumetric flasks were then sonicated for 30 min to ensure complete dissolution and homogeneity.

A known increasing volume of each drug was transferred separately to different stoppered glass vials to obtain solutions within the concentration ranges of (0.25–15.0 mg ml^−1^) for DZP, ALP, CDP, and (0.50–25.0 mg ml^−1^) for ACB. Thereafter, a specific volume of the internal standard (PGC) was inserted per each vial with final concentration of 10.0 mg ml^−1^. After that, the volume of each vial is completed to 1.0 ml using 0.3 M SDS/D_2_O solution. The analyzed samples were prepared by taking 0.5 ml of prepared solutions into NMR tube and experiments were carried out under optimized conditions. A plot of absolute integral areas *vs* concentrations was recorded, and calibration curves were processed.

### Analysis of BDZs in their oral tablet dosage forms

Ten tablets of Valinil^®^ and ten Restolam^®^ tablets containing DZP and ALP were grinded and weighed, respectively. Thereafter, a certain weight of each powdered drug formulation, equivalent to 10.0 and 2.0 mg, respectively was accurately measured and transferred into two separate stoppered glass vials, and 2.0 mls of the 0.3 M SDS/D_2_O solution were added. The prepared stock solutions had final concentrations of 5.0 mg ml^−1^ and 1.0 mg ml^−1^ for DZP and ALP, respectively. After that, sonication was performed for 10 min for the two vials to ensure complete solubility. The above-mentioned procedures employed for constructing the calibration plots were followed and the percentage recovered was measured. Concerning CDP, due to the unavailability of Librium tablets, a synthetic laboratory dosage form was prepared. A stock solution was prepared by dissolving 0.01 g CDP together with starch, lactose, and talc powder in 2.0 mls D_2_O and 0.3 M SDS. The final concentration of the stock solution was 5.0 mg ml^−1^. The analyzed samples were prepared as mentioned before for constructing the calibration curve and recovery percents were determined.

## Results and discussion

**Figure 2:**
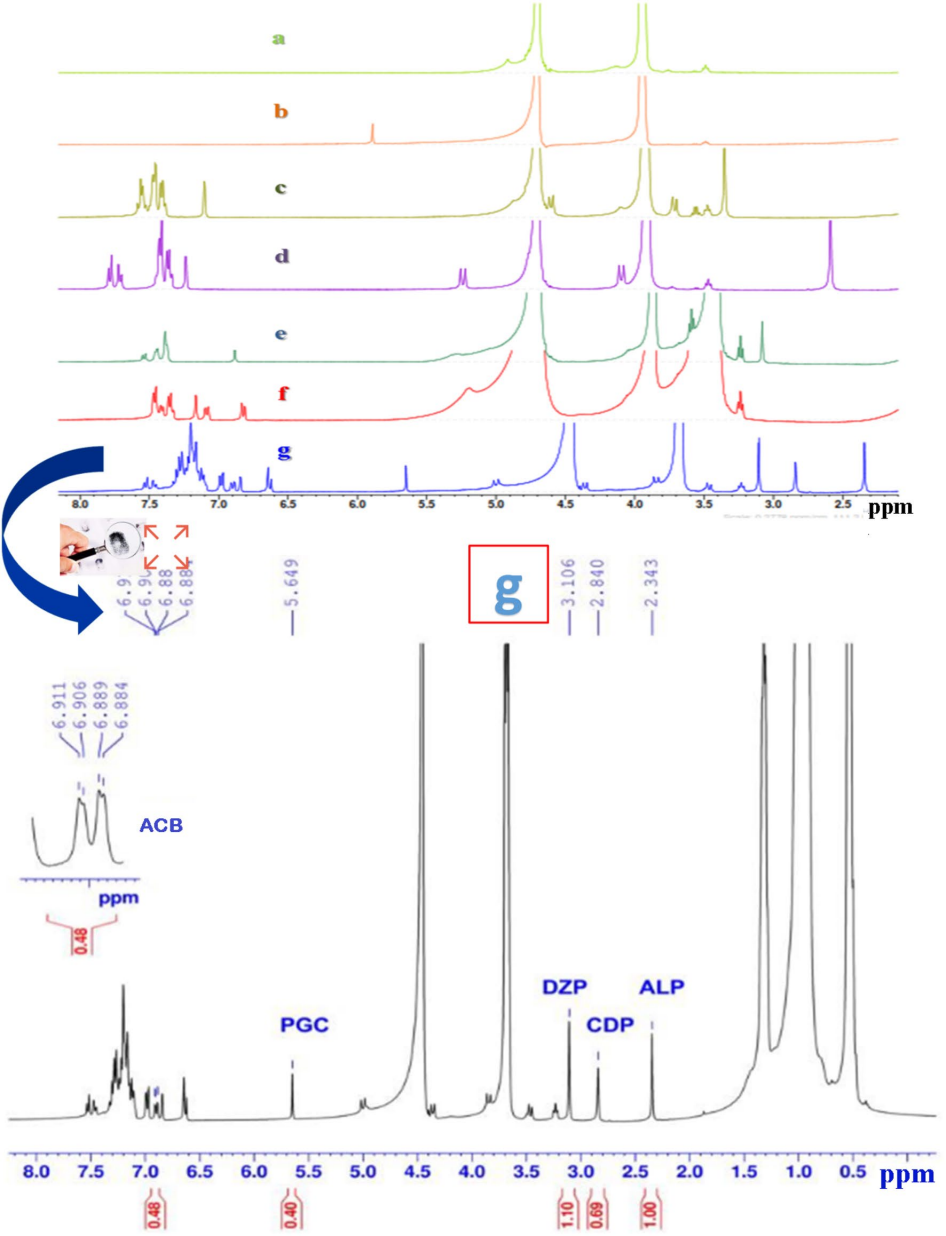
^1^H-qNMR spectra of: (**a**) SDS, (**b**) PGC, (**c**) DZP, (**d**) ALP, (**e**) CDP, (**f**) ACB, and (**g**) the mixture of PGC, DZP, ALP, CDP and ACB under optimized conditions.

In the present study, the conducted spectroscopic ^1^H-qNMR method for assaying the three BDZs (DZP, ALP and CDP) in presence of ACB as an impurity was developed and carefully optimized. Many factors could affect the assaying efficiency by this technique such as: solvent selection, internal standard selection, and different acquisition parameters in the instrument, so all these factors were under consideration, fussily studied, and the most optimum one was utilized. Deuterated water containing 0.3 M SDS was found to be the best-chosen solvent as it dissolved all the analytes with no need for any organic solvents. PGC was used as an internal standard as it didn’t interfere either with BDZs peaks nor ACB impurity peak. PGC produced a well-resolved singlet at 5.65 ppm. The ^1^H-qNMR spectra demonstrated a singlet at 2.35 ppm, 2.84 ppm and 3.11 ppm, referring to ALP, CDP and DZP, respectively. While as, doublet of doublet at 6.91, 6.90, 6.89, 6.88 ppm referring to ACB as shown in Fig. 2.

However, this ^1^H-qNMR method has few limitations related to its low sensitivity compared to HPLC and some other analytical techniques, which is a known disadvantage of qNMR. However, this sensitivity can be improved by increasing the number of scans which may influence the method greenness due to magnetization environmental effect. The expenses of the NMR instrument and its maintenance are other limitations of this method.

### Solvent selection

**Figure 3:**
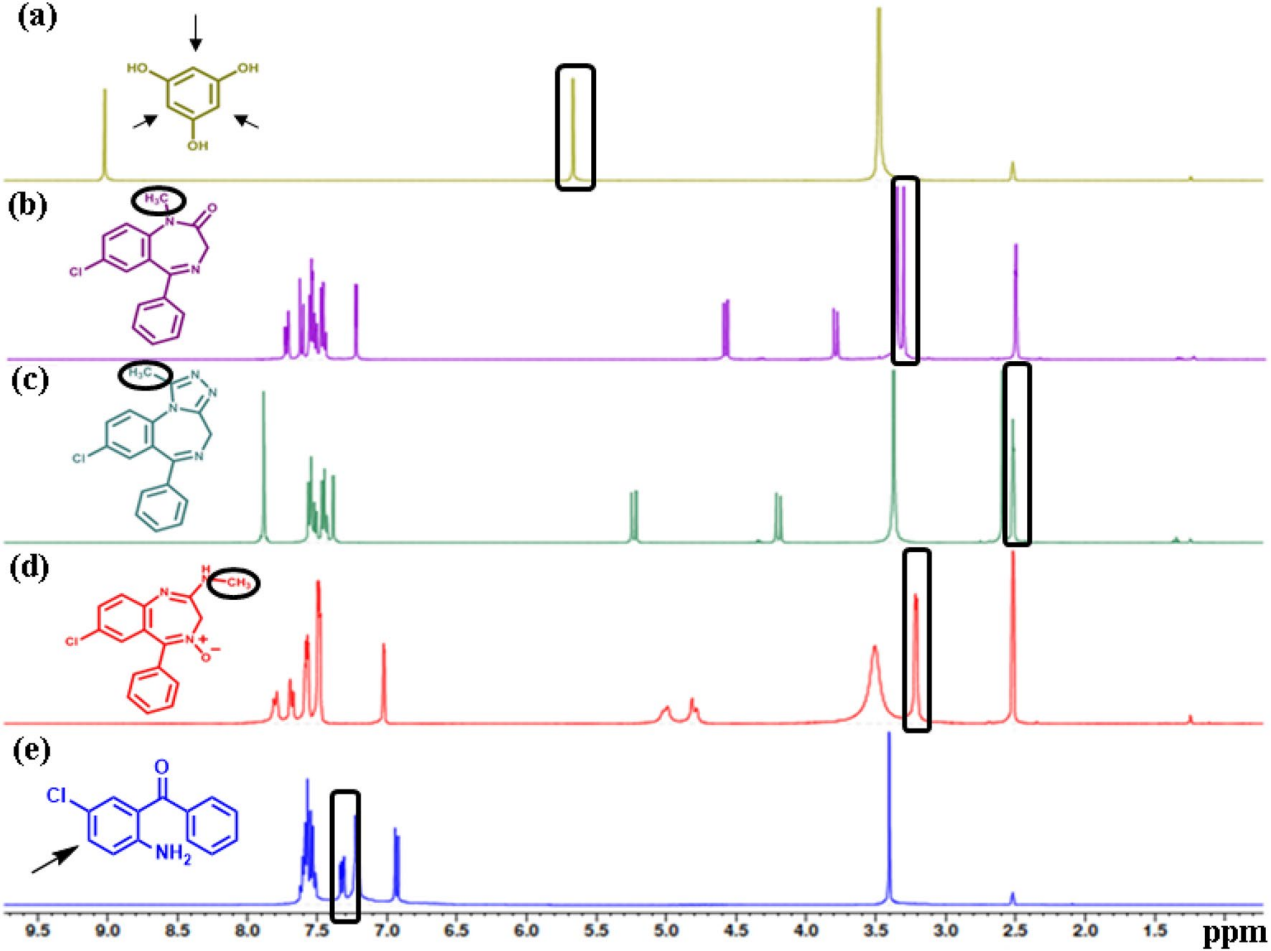
^1^H-qNMR spectra of the mixture: (a) PGC, (b) DZP, (c) ALP, (d) CDP and (e) ACB in DMSO-*d*_*6*_.

Different deuterated solvents were examined to gain the best solubility of all analytes in addition to obtaining the efficient analysis without interference or overlapping of signals. Besides, the greenness concept and maintaining sustainability is a main purpose in this study. Two deuterated solvents were tried including DMSO-*d*_*6*_ and deuterated water (D_2_O). All analytes showed good solubility in DMSO-*d*_*6*_ and their ^1^H-qNMR spectra were shown in Fig. 3. Well-resolved peaks were obtained and proton signals for each compound were detected. Besides, the chemical structures and the purity were illustrated as demonstrated in Fig. 3. To improve the greenness and sustainability of the method, D_2_O was tried instead of DMSO-*d*_*6*_*.* However*,* the poor Solubility of BDZs was problematic^30^. Enhancement of solubility was achieved via two approaches: adding propanol or adding a surfactant like SDS. The best results were obtained via using D_2_O containing SDS as a surfactant rather than D_2_O with propanol. Moreover, SDS is more ecofriendly than propanol, so the utility of it will be more desirable. The concentration of SDS was investigated in the range of (0.06–0.5 M), the lowest concentration that achieved the best solubility without causing supersaturation to the samples was 0.3 M. Therefore, D_2_O solvent containing 0.3 M SDS was chosen as the best system where it allowed the quantitative assay of the BZDs in presence of impurity without any interferences or overlapping of signals with each other or with the used dissolution system. Moreover, it ensured the complete solubility and homogeneity of the analytes.

### Internal standard selection

Many compounds were tested as internal standards such as (pyridoxine, inositol, PGC, and formic acid). PGC was chosen as the best since it showed a singlet at 5.65 ppm with no interference with signals at chemical shifts: 2.35, 2.84, 3.11 and 6.90 ppm, which was used for quantitative assay of ALP, CDP, DZP, and ACB, respectively.

### Technical optimization of NMR conditions

**Figure 4:**
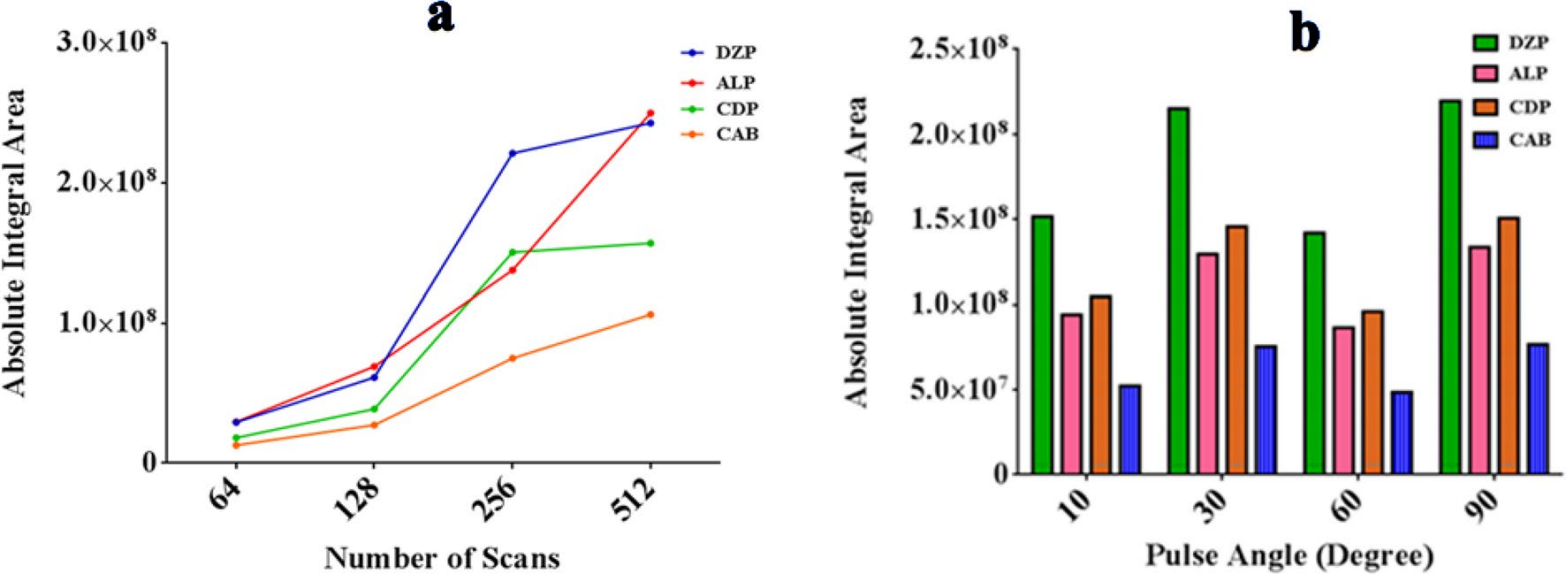
Effect of number of scans (**a**) and pulse angle (**b**) on the absolute integral areas of the selected peaks for the three studied BDZs (DZP, ALP, and CDP) in presence of ACB as impurity.

Many parameters contribute to the efficiency of ^1^H-qNMR technique such as number of scans, pulse angle and a relaxation delay time. Therefore, these parameters were studied to achieve the best sensitivity and resolution results (Fig. 4).

#### Number of scans

The study of scan numbers is crucial for the signal to noise ratio. Therefore, different numbers of scans were studied (64, 128, 256, and 512) to achieve the highest sensitivity. Although scan number 512 yielded the highest sensitivity, high scanning time was consumed, and non-reproducible results were achieved. Hence, scan number 256 was chosen as the optimum scan number for this study as a reproducible high absolute integrated area was achieved in adequately acceptable scanning time with best signal resolution. Each experiment was repeated three times and average results were plotted versus absolute integrated area as shown in Fig. 4a.

#### Pulse angle

Different pulse angles were tried to give satisfactory results. Therefore, angles (10, 30, 60 and 90º) were investigated keeping the scan number 256. The resulting charts revealed that angle 30º yielded the highest resolution and sensitivity as shown in Fig. 4b.

#### Relaxation delay time

One of the most critical factors in ^1^H-qNMR is a relaxation delay time. The best quantitative assay that ensured the perfectly longitudinal relaxation between two adjacent pulses was achieved by adjusting the relaxation delay time to be more than five times the spin–lattice relaxation time (T1) of the selected signals of both the analytes and the internal standard. Spin–lattice relaxation time (T1) is defined as the time required by magnetized molecules to return to normal state^34^, and can be calculated by an inversion recovery experiment.

**Figure 5:**
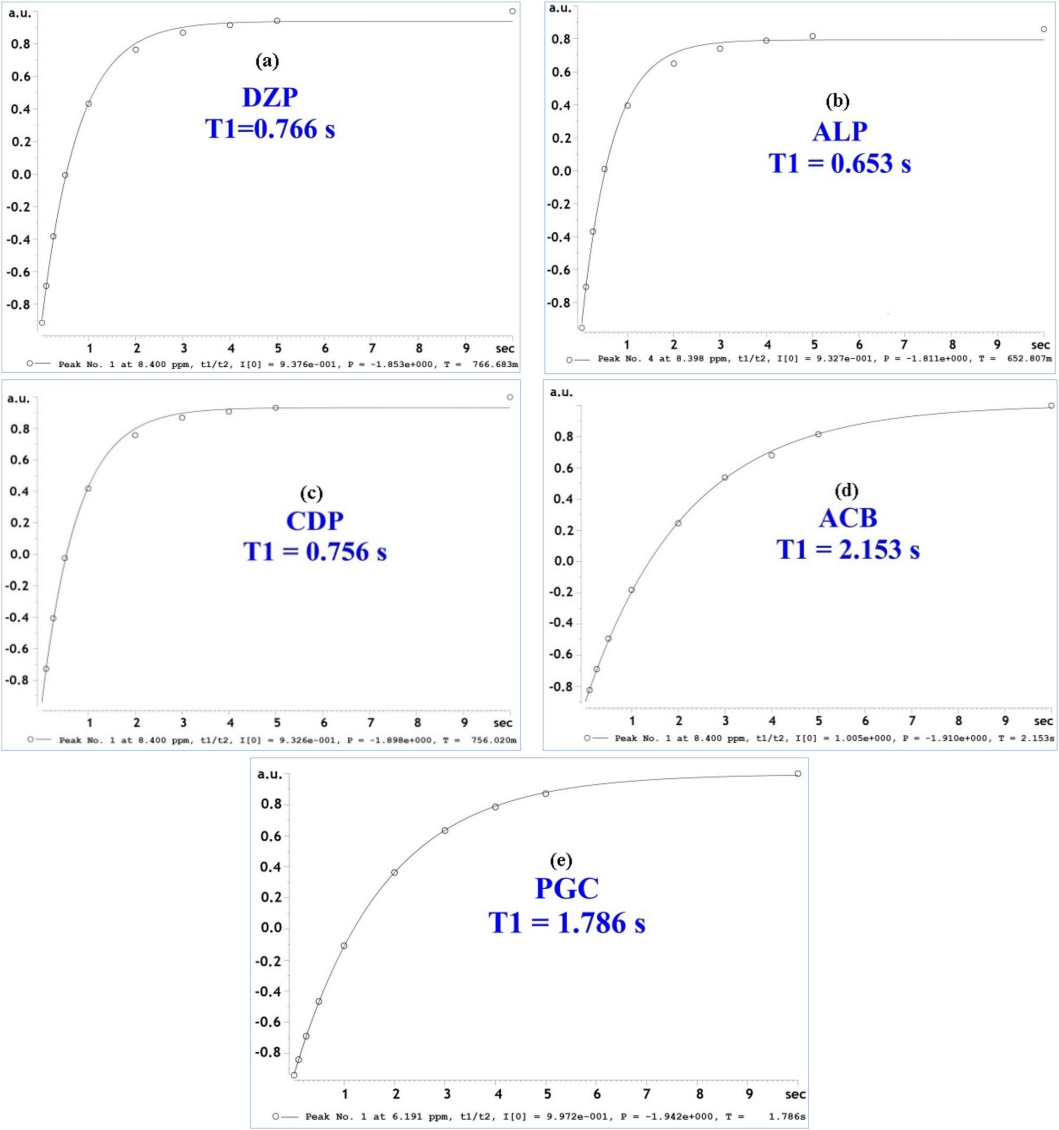
T1 values of (a) DZP, (b) ALP, (c) CDP, (d) ACB and (e) PGC using inversion recovery experiment.

Here in this study, the inversion recovery experiment was performed and T1 spin–lattice relaxation times for DZP, ALP, CDP, ACB and PGC at signals with chemical shifts at 3.11 ppm, 2.35 ppm, 2.84 ppm, 6.90 and 5.65 ppm, respectively were found to be 0.77 s, 0.65 s, 0.77 s, 2.15 s and 1.78 s, respectively. Herein, the best relaxation delay time to be used in this method was found to be 20 s, as it is more than five times the T1 for all signals of interest and is enough to ensure perfectly longitudinal relaxation between two adjacent pulses (Fig. 5). All parameters are listed in Table 1.

**Table 1:** Parameters used for the development of ^1^H-qNMR method of analysis for DZP, ALP, CDP and ACB.

Parameter	Value
Number of scans	256
Pulse angle	30°
Pulse width	4.5 μs
Relaxation Delay time	20 s
Acquisition time	4.08 s
Spectral width	20.02 ppm
Frequency offset	6.175 ppm
Sample temperature	20 °C
Dummy scans	2
Sample spin	On
Data points	65,536

### Method validation

**Figure 6:**
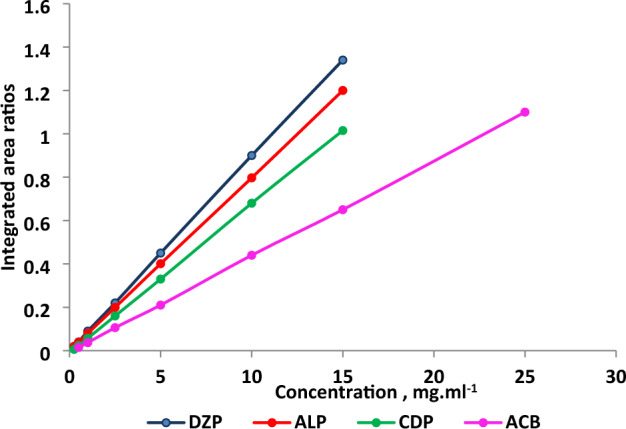
Collected linearity plots of DZP, ALP, CDP and ACB integrated area ratios versus concentration (mg ml^−1^).

The applied method was validated in terms of ICH Q2 (R1)^35^ guidelines for these parameters. A Linear relation between absolute integral area ratio (integral area of the drug and the internal standard; PGC) and concentrations of DZP, ALP, CDP and ACB was illustrated by applying the evolved ^1^H-qNMR method (Fig. 6). The ranges of linearity of DZP, ALP, CDP were found to be (0.25 to 15.0 mg ml^−1^) while the range of linearity of ACB was found to be (0.5–25.0 mg ml^−1^). The results were analyzed and showed a promising linearity with low points scattering, low percentage of relative error (% RE), small value of coefficient of variation (CV) and high value of correlation coefficient (r). The analytical performance data was presented in Table 2.

**Table 2:** Performance data for the determination of DZP, ALP, CDP, and ACB by ^1^H-qNMR method.

Parameter	DZP	ALP	CDP	ACB
Concentration range (mg ml^−1^)	0.25–15.0	0.25–15.0	0.25–15.0	0.5–25.0
Correlation coefficient	0.9999	0. 9999	0.9999	0.9998
Slope	0.09	0.08	0.07	0.04
Intercept	− 0.002	− 8 × 10^–4^	− 0.011	− 0.006
LOD^a^ (mg ml^−1^)	0.06	0.03	0.07	0.16
LOQ^b^ (mg ml^−1^)	0.18	0.09	0.21	0.49
*S*_*y/x*_^c^	0.003	0.001	0.002	0.004
*S*_*a*_^d^	0.001	7 × 10^–4^	0.001	0.002
*S*_*b*_^e^	2 × 10^–4^	1 × 10^–4^	2 × 10^–4^	1 × 10^–4^
CV^f^	1.10	0.97	0.85	1.41
% RE^g^	0.42	0.37	0.32	0.53

^a^Detection limit, ^b^Quantitation limit, ^c^Standard deviation of residuals, ^d^Standard deviation of intercept, ^e^Standard deviation of slop, ^f^Coefficient of variation, ^g^Percentage of relative error.

The limit of detection and quantification of the proposed method mentioned in Table 2 were calculated according to ICH Q2 (R1) specifications^35^ using the following equations:

$${\text{LOD}} = {3}.{3}S_{a} /b$$

$${\text{LOQ}} = {1}0S_{a} /b$$

*S*_*a*_ is the standard deviation of the intercept and *b* is the slope of the calibration curve.

Accuracy is the degree to which the results obtained with the proposed ^1^H-qNMR method corresponds to data obtained via the reference spectrophotometric methods. The accuracy of the proposed method was illustrated using three concentrations of drugs within the linearity range of each drug in pure form and pharmaceutical dosage forms. Concerning DZP, the comparison method depends on the reaction of drug with ammonium thiocyanate (0.05M) and ferric chloride (0.01 M), then the product was measured at 251 nm^31^. On the other hand, ALP and CDP were measured directly at 260 nm and 254 nm, respectively (zero order spectrophotometric methods)^32, 33^. The percent recoveries for the three drugs were calculated and shown in Tables 3 and 4. Results showed that there is no significant difference between the methods by using both Student's *t-*test and variance ratio *F*-test^31^.

**Table 3:** Comparative analytical data for the determination of DZP, ALP, CDP and ACB in pure form by the proposed ^1^H-qNMR method and the reported spectrophotometric methods.

Drug	Proposed method		Reported method^31–33^	
	Amount taken (mg ml^−1^)	Amount found (mg ml^−1^)	% Found^a^	% Found^a^
DZP	0.25	0.25	98.40	98.90
	0.5	0.51	100.40	101.07
	1.0	0.98	98.20	99.63
	2.5	2.47	98.84	
	5.0	5.05	101.02	
	10.0	10.03	100.34	
	15.0	14.97	99.82	
$${\overline{\text{X}}}$$$$\pm$$ SD			99.57 ± 1.10	99.87 ± 1.10
t-test			0.39 (2.23)^b^	
*F*-value			1.01 (8.89)^b^	
ALP	0.25	0.245	98.00	98.06
	0.5	0.494	98.80	102.08
	1.0	1.01	100.80	99.40
	2.5	2.498	99.92	
	5.0	5.021	100.42	
	10.0	9.973	99.73	
	15.0	15.01	100.07	
${\overline{\text{X}}}$ $\pm$ SD			99.68 ± 0.97	99.85 ± 2.05
t-test			0.19 (2.23)^b^	
*F*-value			4.48 (8.89)^b^	
CDP	0.25	0.248	99.20	99.36
	0.5	0.507	101.40	100.63
	1.0	0.991	99.10	99.79
	2.5	2.493	99.72	
	5.0	4.971	99.42	
	10.0	10.073	100.73	
	15.0	14.956	99.71	
$${\overline{\text{X}}}$$$$\pm$$ SD			99.90 ± 0.85	99.93 ± 0.65
t-test			0.05 (2.23)^b^	
*F*-value			1.75 (8.89)^b^	

^a^Each result is the mean recovery of three individual analyses.

^b^he values between brackets are tabulated *t-* and *F*-values at *P* = 0.05.

*The reported spectrophotometric method for DZP ^31^ depended on its reaction with 0.05 M ammonium thiocyanate/0.01 M ferric chloride FeCl_3_ and the product is measure at λ_max_ = 251 nm. The reported method for ALP ^32^ depends on its direct measurement in 0.1 M HCl at λ_max_ = 260 nm. The reported for CDP ^33^, depend on its direct measurement in 0.1 M HCl at 254 nm.

**Table 4:** Intra-day and inter-day precision data for the assay of DZP, ALP, CDP and ACB by the proposed ^1^H-qNMR method.

Parameters		DZP concentration (mg ml^−1^)			ALP concentration (mg ml^−1^)		
		1.0	5.0	10.0	1.0	5.0	10.0
Intra-day	% Found^a^	102.45	100.58	100.34	101.00	100.20	100.10
		100.22	101.69	101.46	99.75	100.70	100.85
		101.33	100.13	99.23	101.13	100.95	99.48
	${\overline{\text{X}}}$	101.33	100.80	100.34	100.63	100.62	100.14
	± SD	1.11	0.80	1.11	0.76	0.38	0.68
	CV	1.09	0.79	1.10	0.75	0.38	0.68
	% Error	0.63	0.46	0.64	0.43	0.21	0.39
Inter-day	% Found^a^	102.45	100.58	100.34	101.00	100.20	100.10
		101.33	99.24	101.57	100.36	101.70	100.35
		99.11	101.02	99.23	99.75	99.70	100.09
	${\overline{\text{X}}}$	100.96	100.28	100.38	100.79	100.53	100.51
	± SD	1.69	0.92	1.16	0.95	1.04	0.72
	CV	1.68	0.92	1.16	0.94	1.03	0.72
	% Error	0.97	0.53	0.67	0.54	0.59	0.41

		CDP concentration (mg ml^−1^)			ACB concentration (mg ml^−1^)		
		2.5	5.0	10.0	5.0	10.0	25.0
Intra-day	% Found^a^	99.71	99.42	100.37	98.37	101.34	100.40
		100.29	101.17	99.27	100.63	102.47	101.31
		99.13	100.29	100.00	99.27	101.79	99.49
	${\overline{\text{X}}}$	99.71	100.29	99.76	99.43	101.87	100.40
	± SD	0.58	0.87	1.11	1.14	0.57	0.90
	CV	0.58	0.87	0.12	1.14	0.56	0.90
	% Error	0.34	0.50	0.64	0.66	0.32	0.52
Inter-day	% Found^a^	99.71	99.42	100.37	98.37	101.34	100.40
		98.54	98.83	101.46	100.18	101.79	102.21
		101.46	101.46	99.56	97.91	99.75	99.95
	${\overline{\text{X}}}$	99.90	99.90	100.58	98.82	100.96	100.85
	± SD	1.46	1.37	0.95	1.19	1.07	1.20
	CV	1.46	1.37	0.95	1.21	1.06	1.19
	% Error	0.84	0.79	0.54	0.70	0.61	0.68

^a^Each result is the mean recovery of three individual analyses.

The intra-day and inter-day precision of proposed ^1^H-qNMR method were estimated by the assay of DZP, ALP, CDP and ACB three times using three different concentrations in one day and for three successive days. The results showed low values of relative standard deviations which confirmed the precision and good reproducibility of the proposed method as shown in Table 4**.**

### Specificity and selectivity

**Figure 7:**
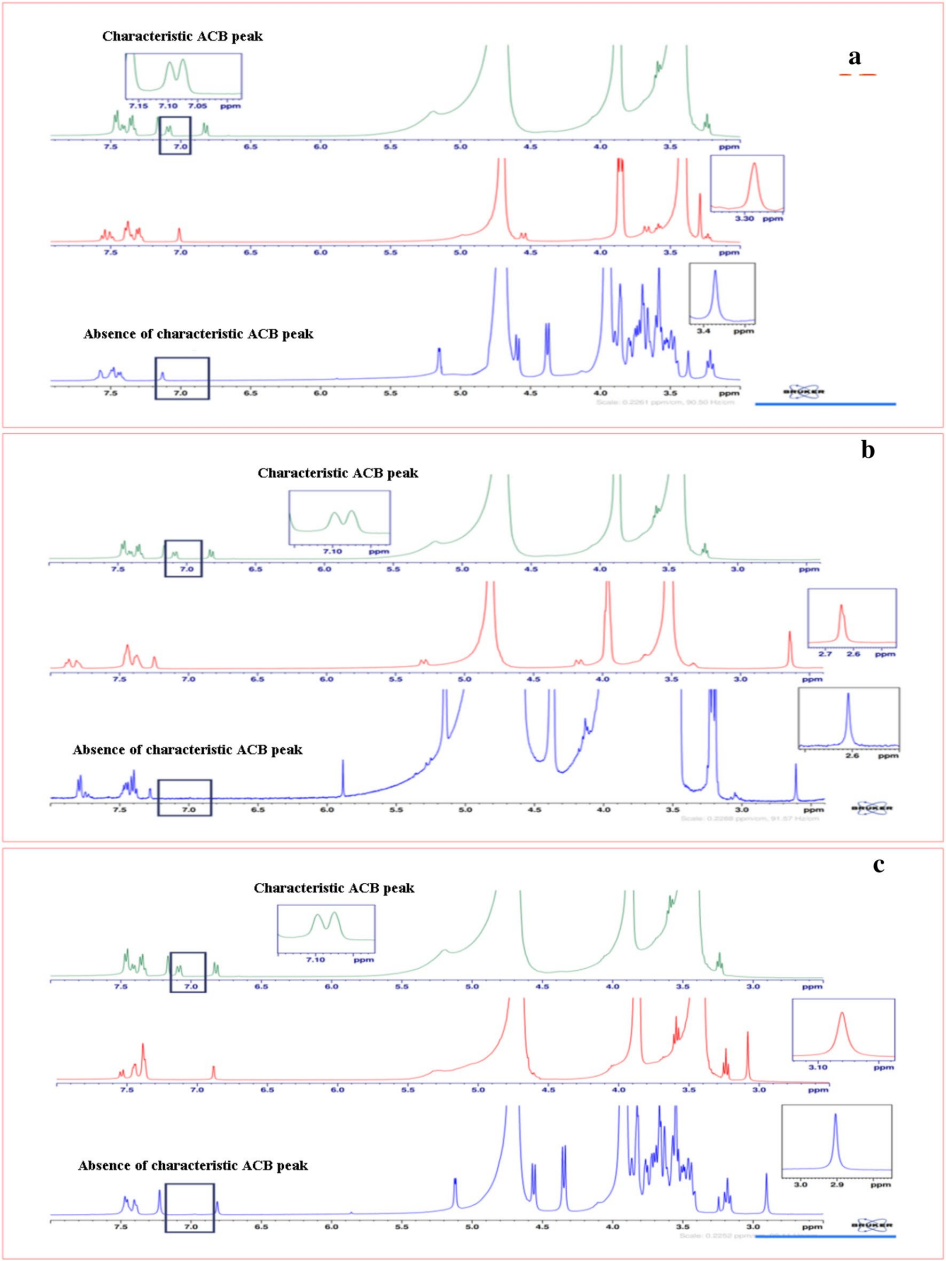
A typical ^1^H-qNMR spectra of (**a**) DZP, (**b**) ALP and (**c**) CDP in both pure form and tablet dosage form with proof the absence of ACB impurity. Where, the green color represents ^1^H-qNMR spectrum of ACB, red color represents the ^1^H-qNMR spectra of drugs in pure form while blue color represents the ^1^H-qNMR spectra of drugs in tablet dosage form.

The specificity and selectivity of the applied proposed method were evaluated to emphasize the absence of any negative impact of excipients, dissolution system or an impurity on the signals of interest. The evolved method proved the high specificity and selectivity as it was capable of simultaneous determination of three BDZs (DZP, ALP and CDP) in presence of their impurity ACB without any interference with each other in both pure form and pharmaceutical dosage forms as appeared in Fig. 2g and Fig. 7.

### Robustness

Robustness means measuring the capability of the method to keep unchanged by small intentional variations in the method parameters, and gives an indication of method reliability through normal operation^36, 37^. The method's robustness was evaluated by varying the studied parameters; pulse angle (30–32), relaxation delay (20–22 s), and number of scans (256–258), and check its effect on results. The variations of different studied NMR parameters showed little effect on the measured response as shown by the excellent recovery% and small values of SD (Table 5) which verifies the method robustness.

**Table 5:** Robustness estimation for DZP, ALP, CDP and ACB using the proposed ^1^H-qNMR method.

Tested parameter		No. of scans (256–258)	Pulse angle (30–32)	Relaxation delay time (20–22 s)
DZP	Mean recovery%	100.74	103.7	98.52
	SD	0.01	0.007	0.014
ALP	Mean recovery%	98.35	99.17	101.65
	SD	0.01	0.01	0.02
CDP	Mean recovery%	98.04	106.86	102.94
	SD	0.01	0.05	0.02
ACB	Mean recovery% SD	99.09	101.82	100.90
		0.02	0.014	0.007

### Application

The applied method was adopted for the determination of three BDZs (DZP, ALP and CDP) in presence of ACB impurity in bulk and pharmaceutical dosage forms. Three different concentrations of each drug within linearity range were used. The observed results were precise and in agreement with labeled claim as indicated by low percentage value of relative standard deviation and high percentage value of purity as shown in Table 6. The results were statistically analyzed and compared with other comparison spectrophotometric methods^31–33^ using variance ratio *F*-test and student’s.

**Table 6:** Comparative analytical data for the determination of DZP, ALP and CDP in their pharmaceutical dosage forms by the proposed ^1^H-qNMR method and reported spectrophotometric methods.

Drug	DZP	ALP		
	Proposed method	Reported method^31^	Proposed method	Reported method^32^
${\overline{\text{X}}}$_a_ $\pm$ SD	99.87 ± 1.10	99.87 ± 1.10	99.85 ± 2.04	99.24 ± 1.41
t-test	0.25 (2.23)^b^	0.42 (2.23)^b^		
*F*-value	1.84 (8.89)^b^	2.09 (8.89)^b^		

Drug	CDP			
	Proposed method	Reported method^33^		
${\overline{\text{X}}}$_a_ $\pm$ SD	99.93 ± 0.65	99.88 ± 1.68		
t-test	0.05 (2.23)^b^			
*F*-value	1.47 (19)^b^			

^*a*^ Each result is the mean recovery of three individual analyses.

^*b*^The values between brackets are tabulated *t-* and *F*-values at *P* = 0.05.

*The reported spectrophotometric method for DZP^31^ depended on its reaction with 0.05 M ammonium thiocyanate/0.01 M FeCl_3_ and the product is measure at λ_max_ = 251 nm. The reported method for ALP^32^ depends on its direct measurement in 0.1 M HCl at λ_max_ = 260 nm. The reported for CDP^33^, depend on its direct measurement in 0.1 M HCl at 254 nm.

t-test^38^. The results showed that there is no significant difference and reflected the applicability of ^1^H-qNMR spectroscopic method for quality control of dosage forms.

### Assessment of greenness

The application of green methods in assaying different compounds is currently attracting interest in research. Therefore, the evaluation and assessment of the green methods is crucial too. Four different methods were applied to assess the performed analytical green ^1^H-qNMR method as well as comparing their green performance with that of the three reported spectrophotometric methods^31–33^. The reported spectrophotometric method for DZP^31^ depended on its reaction with 0.05 M ammonium thiocyanate/0.01 M FeCl_3_ and the product is measure at λ_max_ = 251 nm. The reported method for ALP^32^ depends on measuring its UV absorbance in 0.1 M HCl at λ_max_ = 260 nm. The reported for CDP^33^, depend on the measurement of its absorbance in 0.1 M HCl at 254 nm.

#### Assessment of greenness using Analytical Eco-Scale

In this metric, the solvents used, energy consumption, hazardous conditions, and method processing were evaluated for their greenness. This assessment is based on calculating the penalty points for the proposed analytical ^1^H-qNMR method by multiplying the sub-total penalty points for a certain volume and hazard, then subtracting the penalty points from a base 100. If the proposed method has *Eco-Scale* value more than 75 then it is considered as excellent green method. Moreover, if *Eco-Scale* value was found more than 50, the method is considered as accepted. However, if the *Eco-Scale* value is less than 50, then the method is inconvenient^39–41^. For the proposed method, the *Eco-Scale* value was estimated to be 97 compared to the *Eco-Scale* values of the reported spectrophotometric methods^31–33^ which are 86, 84 and 88, respectively. The obtained *Eco-Scale* value reveals that the proposed method is an excellent green procedure for the analysis of the studied drugs as shown in Table 7.

**Table 7:** Analytical comparison between the proposed ^1^H-NMR method and the reported spectrophotometric methods by applying analytical Eco-Scale approach.

	Penalty points			
	Proposed ^1^H-qNMR method	Reported spectrophotometric methods*		
		^31^	^32^	^33^
Reagents				
SDS	0	–	–	–
Hydrochloric acid	–	6	6	6
Ferric chloride	–	1	–	–
Ammonium thiocyanate	–	1	–	–
Ethanol	–	–	4	–
Deuterated water	0	–	–	–
Instruments				
Energy	2	0	0	0
Occupational hazard	0	3	3	3
Waste	1	3	3	3
Total penalty points	3	14	16	12
Analytical *Eco-Scale* (Total score)	97	86	84	88

*The reported spectrophotometric method for DZP ^31^ depended on its reaction with 0.05 M ammonium thiocyanate/0.01 M FeCl_3_ and the product is measure at λ_max_ = 251 nm. The reported method for ALP ^32^ depends on its direct measurement in 0.1 M HCl at λ_max_ = 260 nm. The reported for CDP ^33^, depend on its direct measurement in 0.1 M HCl at 254 nm.

#### Assessment of greenness using green analytical procedure index (GAPI)

The GAPI uses five pentagrams to assess and measure the environmental hazards of every step during the analytical process. Green, yellow and red colors reveal low, medium and high effect on the environmental^42, 43^, respectively. The GAPI assessment for the proposed method showed green character in ten fields, yellow in one and one red field which shows that performed method has the privilege more than the other reported methods^31–33^ as it satisfies many green requirements as shown in Table 8.

**Table 8:** Analytical comparison between the proposed ^1^H-NMR method and the reported spectrophotometric methods by applying GAPI and AGREE approaches.

^1^H-qNMR method	Reported Spectrophotometric methods		
DZP, ALP and CDP	DZP^31^	ALP^32^	CDP^33^
GAPI			
			
AGREE			
			

*The reported spectrophotometric method for DZP ^31^ depended on its reaction with 0.05 M ammonium thiocyanate/0.01 M FeCl_3_ and the product is measure at λ_max_ = 251 nm. The reported method for ALP ^32^ depends on its direct measurement in 0.1 M HCl at λ_max_ = 260 nm. The reported for CDP ^33^, depend on its direct measurement in 0.1 M HCl at 254 nm.

#### Assessment of greenness using analytical greenness metric approach (AGREE)

Green analytical chemistry targets the use of eco-friendly procedures with higher safety towards the environment and humans. In the AGREE approach, twelve principles are assessed to give a final score ranging from zero to one. The closer to score one, the more green is the used method^44, 45^. Applying AGREE method and its parameters for the proposed ^1^H-qNMR method and the three reported spectrophotometric methods ^31–33^, it was found that the proposed ^1^H-qNMR has the highest score of 0.82 compared to the reported methods^31–33^ with scores of 0.65, 0.63 and 0.66 , respectively as shown in Table 8.

#### Assessment of the method whiteness using RGB additive color model

Assessment of whiteness of the developed qNMR analytical method was also done by using RGB model which is considered an extension for greenness assessment of analytical method^46^. Whiteness assessment combines the assessment of the method’s REDNESS (analytical performance), GREENNESS (safety and eco-friendliness) and BLUENESS (productivity/practical effectiveness)^46–48^.

Each color is evaluated for selected criteria; accuracy (%), linearity range, limit of quantification (LOQ) and inter-day precision (CV values) are the selected criteria for redness assessment, liquid chemicals consumption, chemicals safety/hazards, additional occupational risk factors and energy intake are the selected criteria for greenness assessment, and cost of analysis, instrument technical service frequency, destruction of sample material and time of analysis are the selected criteria for blueness assessment.

**Figure 8:**
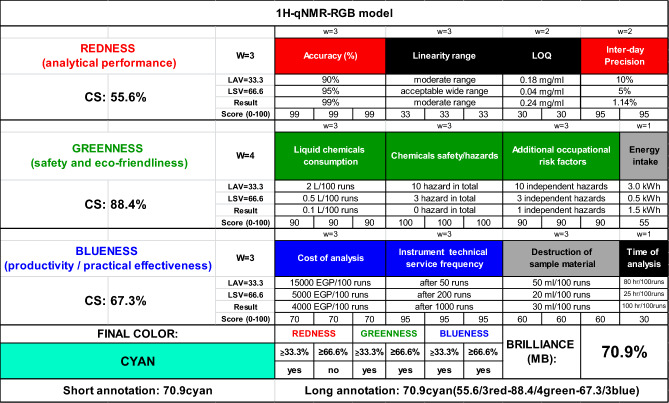
RGB Additive Color Model for analysis of whiteness of the proposed ^1^H-qNMR method.

It was found that the proposed ^1^H-qNMR method has red color score (CS%) of 55.6%, green color score (CS%) of 88.4% and blue color score (CS%) of 67.3%. Therefore, the method brilliance (MB), the qualitative evaluation of method whiteness, was 70.9%, and the final color was cyan that proves the greenness and practical effectiveness of the used qNMR analytical method as shown in Fig. 8.

Comparatively in the reported spectrophotometric methods^31–33^, Fig. S1–S3 showed that the proposed ^1^H-qNMR has better greenness and safety profile, practical effectiveness, and better combined whiteness (MB) score compared to the reported spectrophotometric method.

## Conclusion

A green, ecological, precise, rapid, and efficient ^1^H-qNMR method was developed for the concurrent determination of three BDZs (DZP, ALP and CDP) in presence of ACB as a common impurity in BDZs. It is the first time to utilize a surfactant like SDS in a dissolution system with deuterated water to be used as a solvent in NMR technique instead of the common NMR solvents. The conducting method was fully validated and successfully applied to estimate BDZs in raw materials, and dosage forms, using phloroglucinol as the internal standard. The proposed method can be readily applied for purity check of the three BDZs. The designed method exhibits many advantages by virtue of its sustainability and greenness due to the absence of organic solvents and toxic chemicals. Furthermore, it is an efficient method not only for multiple analytes determination per run but also for detection of impurity in the meantime. This method provides an opportunity to develop greener ^1^H-qNMR analytical methods using eco-friendly systems utilizing aqueous solutions of surfactants only. The application of an experimental design for method optimization and development is recommended for future applications. This will further enhance the efficiency of the method.

### Supplementary Information

Supplementary Figures.

## Data Availability

The datasets used and/or analyzed during the current study are available from the corresponding author on reasonable request.
