# Supplementary material for: Utility and greenness appraisal of nuclear magnetic resonance for sustainable simultaneous determination of three 1,4-benzodiazepines and their main impurity 2-amino-5-chlorobenzophenone
Source: Sci Rep. 2023 Nov 30;13:21121. doi: 10.1038/s41598-023-48416-7 (PMC10689731; doi:10.1038/s41598-023-48416-7)
Supplement: Supplementary file 1 — Supplementary Figures. [file 41598_2023_48416_MOESM1_ESM.docx]

**Utility and Greenness Appraisal of Nuclear Magnetic Resonance for Sustainable Simultaneous Determination of three 1,4-Benzodiazepines and their Main Impurity 2-Amino-5-Chlorobenzophenone**

**Nermeen A. Qandeel^a*^,** **Amal A. El-Masry^a^, Rania El-Shaheny^b^_,_** **Manal Eid^b^,** **Mohamed A. Moustafa^a^**

^a^Department of Medicinal Chemistry, Faculty of Pharmacy, Mansoura University, Mansoura 35516, Egypt

^b^Department of Pharmaceutical Analytical Chemistry, Faculty of Pharmacy, Mansoura University, Mansoura 35516, Egypt

*Corresponding author: Department of Medicinal Chemistry, Faculty of Pharmacy, Mansoura University, Mansoura 35516, Egypt, [N.qandeel@mans.edu.eg](mailto:N.qandeel@mans.edu.eg), Phone Number: +201062624029

|  |
| --- |

^31^

**Fig. S1.** RGB Additive Color Model for analysis of whiteness of the reported spectrophotometric method of DZP.

^32^

**Fig. S2.** RGB Additive Color Model for analysis of whiteness of the reported spectrophotometric method of ALP.

^33^

**Fig. S3.** RGB Additive Color Model for analysis of whiteness of the reported spectrophotometric method of CDP.
